# Measuring Tumor Metabolism in Pediatric Diffuse Intrinsic Pontine Glioma Using Hyperpolarized Carbon-13 MR Metabolic Imaging

**DOI:** 10.1155/2018/3215658

**Published:** 2018-07-30

**Authors:** Adam W. Autry, Rintaro Hashizume, C. David James, Peder E. Z. Larson, Daniel B. Vigneron, Ilwoo Park

**Affiliations:** ^1^Department of Radiology and Biomedical Imaging, University of California, San Francisco, CA, USA; ^2^Department of Neurological Surgery and Biochemistry and Molecular Genetics, Northwestern University, Chicago, IL, USA; ^3^Department of Radiology, Chonnam National University Medical School, Gwangju, Republic of Korea; ^4^Department of Radiology, Chonnam National University Hospital, Gwangju, Republic of Korea

## Abstract

**Objective:**

The purpose of this study was to demonstrate the feasibility of using hyperpolarized carbon-13 (^13^C) metabolic imaging with [1-^13^C]-labeled pyruvate for evaluating real-time *in vivo* metabolism of orthotopic diffuse intrinsic pontine glioma (DIPG) xenografts.

**Materials and Methods:**

3D ^13^C magnetic resonance spectroscopic imaging (MRSI) data were acquired on a 3T scanner from 8 rats that had been implanted with human-derived DIPG cells in the brainstem and 5 healthy controls, following injection of 2.5 mL (100 mM) hyperpolarized [1-^13^C]-pyruvate.

**Results:**

Anatomical images from DIPG-bearing rats characteristically exhibited T_2_-hyperintensity throughout the cerebellum and pons that was not accompanied by contrast enhancement. Evaluation of real-time *in vivo*^13^C spectroscopic data revealed ratios of lactate-to-pyruvate (*p* < 0.002), lactate-to-total carbon (*p* < 0.002), and normalized lactate (*p* < 0.002) that were significantly higher in T_2_ lesions harboring tumor relative to corresponding values of healthy normal brain. Elevated levels of lactate in lesions demonstrated a distinct metabolic profile that was associated with infiltrative, viable tumor recapitulating the histopathology of pediatric DIPG.

**Conclusions:**

Results from this study characterized pyruvate and lactate metabolism in orthotopic DIPG xenografts and suggest that hyperpolarized ^13^C MRSI may serve as a noninvasive imaging technique for *in vivo* monitoring of biochemical processes in patients with DIPG.

## 1. Introduction

Diffuse intrinsic pontine glioma (DIPG) comprises a heterogeneous class of childhood brainstem cancers that defy molecular stratification and surgical intervention because of their sensitive location. After forming in the pons, this relatively inaccessible disease often undergoes rapid growth that is characterized by diffuse infiltration across the midline through healthy tissue. Despite decades of clinical trials investigating the efficacy of novel treatment regimens, DIPG remains the leading cause of death among pediatric patients with central nervous system cancers and carries an average survival of only 9 months [1].

Magnetic resonance imaging (MRI) serves as the standard modality for diagnosing DIPG and monitoring disease status in response to treatment [2]. While conventional MRI provides information regarding anatomical changes, its prognostic value and ability to assess physiologic or functional alterations associated with therapeutic efficacy are highly limited [3]. Attempts at using positron emission tomography to evaluate DIPG metabolism have also presented challenges owing to the risks of ionizing radiation exposure [4, 5]. Given the heterogeneous presentation of the disease on imaging, which lacks features for stratifying aggressiveness [6], the development of noninvasive metabolic imaging methods may enhance the evaluation of molecular characteristics as well as response to therapy.

Representing a novel advance in solid state physics, dissolution dynamic nuclear polarization (d-DNP) enables the acquisition of carbon-13 (^13^C) magnetic resonance data with an appreciable gain in sensitivity over conventional methods [7]. A phase I first-in-human study using hyperpolarized ^13^C magnetic resonance spectroscopic imaging (MRSI) has already demonstrated the safety and feasibility of such technology for probing real-time metabolism in prostate cancer patients [8]. The purpose of this study was to explore the feasibility of using hyperpolarized ^13^C metabolic imaging with [1-^13^C]-pyruvate for evaluating real-time *in vivo* metabolism of orthotopic DIPG xenografts.

## 2. Materials and Methods

Eight six-week-old male athymic rats (rnu/rnu, homozygous, and median weight = 290 g) purchased from Harlan (Indianapolis, IN) were implanted with patient-derived human DIPG cells (SF8628) in the brainstem to create an orthotopic DIPG model, while 5 healthy rats served as experimental controls. The details of the cell culture and intracranial implantation procedures have been described elsewhere [9]. Study procedures were approved by the Institutional Animal Care and Use Committee.

All animals were scanned on a 3T clinical MRI system (GE Healthcare, Waukesha, WI, USA) equipped with a custom-designed ^1^H/^13^C rat coil on approximately the 58th day after tumor implantation. The body temperature was maintained using a heated pad positioned inside the RF coil. Anesthesia was maintained with a constant delivery of 1.5% isoflurane. For the polarization of pyruvate, a 35 *μ*L of [1-^13^C]-pyruvate mixed with 15 mM OX063 trityl radical (GE Healthcare, Oslo, Norway), and 1.5 mM gadolinium (Gd)-DOTA was polarized using a HyperSense® (Oxford Instruments, Abingdon, UK) [7, 10]. After 60 minutes of microwave irradiation, the mixture was dissolved in a saline solution with 5.96 g/L Tris (40 mM), 4.00 g/L NaOH (100 mM), and 0.1 mg/L Na_2_ ethylenediaminetetraacetic acid. The final solution had a concentration of 100 mM pyruvate, and pH∼7.5. 2.7 mL of the dissolved pyruvate solution was injected into the tail vein of the rats over 10 s duration.

The following ^1^H and ^13^C data were acquired in sequence for each scan: (1) axial T_2_-weighted images using a fast spin-echo sequence (echo time/repetition time = 60/4000 ms, 8 cm field of view, 256 × 256 matrix, and 2 mm slice thickness), (2) compressed-sensing ^13^C 3D MRSI data (echo time/repetition time = 140/215 ms, phase encoding in *x* and *y* axes, flyback echo-planar readout in *z*-axis, 20 × 16 × 16 matrix, and 2 × 2 × 5.4 mm spatial resolution) [11] acquired at 20 s from the start of the pyruvate injection, and (3) axial T_1_-weighted images using a spin-echo sequence (echo time/repetition time = 10/700 ms, 8 cm field of view, 320 × 192 matrix, and 1.2 mm slice thickness) after the injection of 0.2 mmol/kg Gd-DTPA.

**Figure 1:**
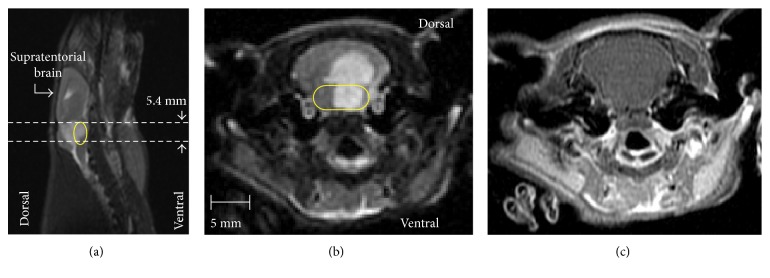
Representative anatomical images from a rat (*n*=8 total) bearing orthotopic DIPG. T_2_-weighted images in sagittal (a) and axial (b) planes demonstrated a T_2_-hyperintense lesion through the brainstem. The corresponding axial post-Gd T_1_-weighted image (c) exhibited no contrast enhancement. Horizontal dashed lines in (a) delimit the 5.4 mm axial slice of ^13^C MRSI data, presented in Figure 2. The yellow boundary in (a) and (b) indicates the location of pons.

The methods for processing ^13^C MRSI data have been described previously [11]. For quantification of ^13^C metabolites, the ratio of lactate-to-pyruvate and lactate-to-total carbon (tC: sum of lactate, pyruvate-hydrate, alanine, and pyruvate) was calculated. In addition, the lactate and pyruvate signals in the brainstem were normalized with respect to the relative signals of the normal brain in the supratentorial region (Figure 1(a)). ROIs were manually contoured on T_2_-weighted images for the T_2_-hyperintense lesion, and the percentage of T_2_ lesion volume was calculated for each voxel. Comparison of mean ^13^C parameters from the T_2_-hyperintense lesion (voxels with >75% T_2_ lesion) and similar region of the infratentorial brain of healthy control animals was performed using the Mann–Whitney rank-sum test. In order to evaluate the spatial variation of ^13^C metabolites, the T_2_-hyperintense lesion was also compared with the contralateral brain (voxels with nonhyperintense tissue on the opposite side of the T_2_ lesion).

Immediately following the scan, tumor-bearing animals were sacrificed and harvested for their brains, which were fixed in phosphate-buffered 4% formalin. Samples were then dehydrated by graded ethanol and embedded in Paraplast Plus wax (McCormick Scientific). 5 *µ*m sections were examined following haematoxylin and eosin (H&E) staining.

## 3. Results and Discussion

Representative anatomical data from a rat injected with DIPG cells are shown in Figure 1, with panel (a) providing an overview of an orthotopic lesion around the brainstem on a sagittal T_2_-weighted image. The corresponding axial T_2_-weighted image exhibited hyperintensity throughout the cerebellum and pons (Figure 1(b)), while no contrast enhancement was visible from the post-Gd T_1_-weighted image acquired at the same location (Figure 1(c)).

**Figure 2:**
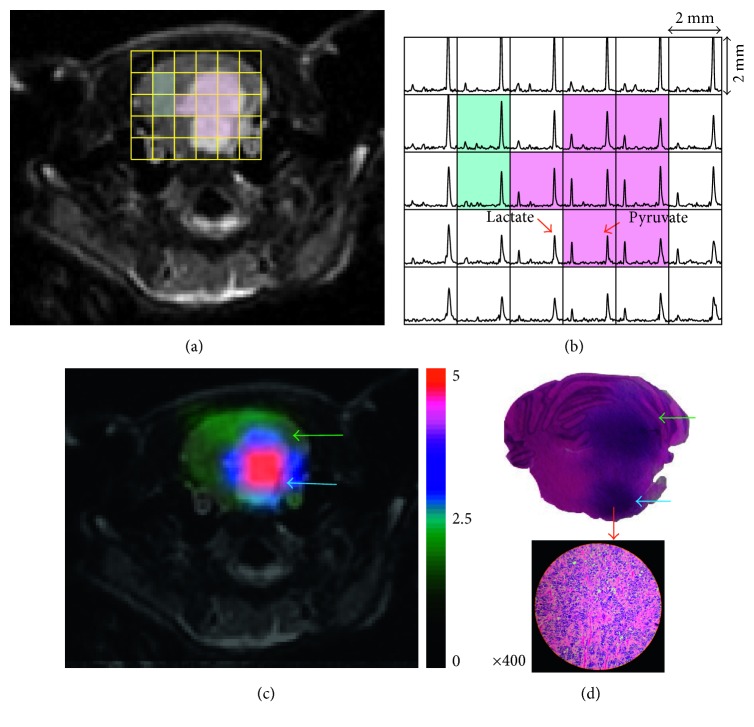
Representative hyperpolarized ^13^C data from a rat (*n*=8 total) bearing DIPG. (a) Axial T_2_-weighted image of the brainstem with a spectral grid overlay for 3D ^13^C MRSI data. Pink and light blue boxes represent voxels encompassing the T_2_-hyperintense lesion and contralateral brain tissue, respectively. (b) The corresponding hyperpolarized ^13^C spectra with an in-plane spatial resolution of 2 × 2 × 5.4 mm^3^. Voxels encompassing the T_2_-hyperintense lesion (pink boxes) exhibited elevated lactate levels compared to those of the contralateral brain tissue (light blue boxes). (c) The map of normalized lactate shows elevated lactate signal in DIPG. (d) The corresponding slice from H&E staining (top) and the zoomed-in H&E image (bottom) demonstrate infiltrative, viable DIPG. The blue and green arrows in (c) and (d) indicate pons and cerebellum of the rat brain, respectively.

The axial T_2_-weighted image in Figure 2(a) depicts a ^13^C MRSI grid superimposed over the brainstem. The corresponding hyperpolarized ^13^C spectra from the same animal (Figure 2(b)) demonstrated the spatial distribution of high ^13^C-labeled lactate and pyruvate signals over the entire brainstem region. Illustrative of the full cohort, these hyperpolarized ^13^C MRSI data demonstrated levels of lactate in the T_2_ lesions (pink voxels; Figures 2(a) and 2(b)) that were elevated relative to the contralateral normal brain (blue voxels; Figures 2(a) and 2(b)).

Metabolite parameters derived from hyperpolarized data are compared between DIPG xenografts and healthy control brains in Table 1. The T_2_-hyperintense tumors exhibited highly elevated metabolism compared to both healthy controls and the contralateral hemisphere, which may contain infiltrating tumor. The ratios of lactate-to-pyruvate, lactate-to-total carbon, and normalized lactate in T_2_ lesions (0.70 ± 0.24, 0.36 ± 0.08, and 2.9 ± 1.1, resp.) were significantly higher than the corresponding values in the healthy normal brain (0.20 ± 0.06, 0.14 ± 0.03, and 1.1 ±0.25, resp.). The normalized lactate map in Figure 2(c) shows the differential production of lactate between the DIPG xenograft and tissue in the contralateral hemisphere. From the corresponding H&E-stained slice, there was observed infiltrative, viable tumor that recapitulated the histopathology of pediatric DIPG (Figure 2(d)). In contrast, the normalized pyruvate was found to be similar across both regions and comparable to the healthy brain (Table 1).

**Figure 3:**
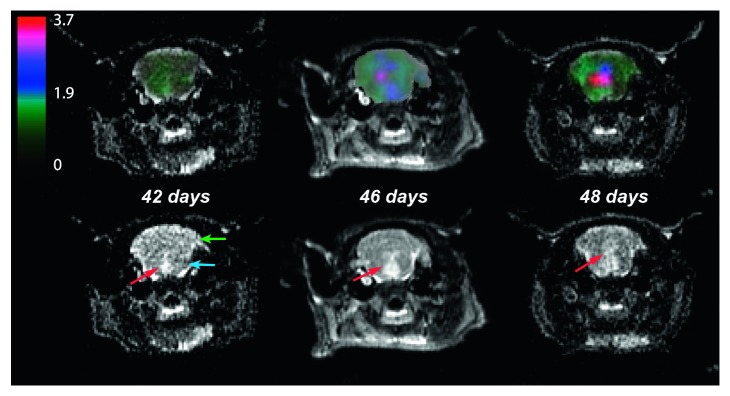
Longitudinal ^13^C spectral data showing normalized lactate from a rat imaged at 42, 46, and 48 days from implantation (left to right). The top row displays the map of normalized lactate overlaid on the same T_2_-weighted images of the bottom row. These data show a several-fold increase in the metabolic abnormality associated with the evolution of the anatomic lesion. The red arrows indicate T_2_ lesion in the brainstem, while blue and green arrows indicate pons and cerebellum of the rat brain, respectively. The tumor initially developed in the pons and diffused across the cerebellum.

In order to assess the ability to observe longitudinal changes in metabolism, hyperpolarized ^13^C MRSI data were obtained from an additional single animal imaged over a period of 7 days in the course of tumor development (Figure 3). The longitudinal change in normalized lactate signal and T_2_-hyperintensity are shown in Figure 3. Normalized lactate from the ^13^C spectral data imaged at 42, 46, and 48 days from implantation were 1.2 ± 0.2, 2.5 ± 0.1, and 3.5 ±1.0, respectively. These data show a severalfold increase in the metabolic abnormality associated with the evolution of the anatomic lesion.

This study has demonstrated the feasibility of using hyperpolarized ^13^C metabolic imaging to assess *in vivo* metabolism in orthotopic brainstem xenografts that contain patient-derived primary DIPG cells. By using hyperpolarized [1-^13^C]-pyruvate in conjunction with rapid 3D MRSI acquisition techniques, it was shown that nonenhancing brainstem glioma can be evaluated on the basis of real-time molecular data, as an initial step towards noninvasive disease characterization. To the best of our knowledge, this is the first study to apply hyperpolarized ^13^C techniques in brainstem tumor as well as in nonenhancing brain tumor.

An important feature of the orthotopic murine tumor model adopted here was its ability to recapitulate aspects of disease observed in patients. With regard to imaging, the longitudinal data revealed a similar pattern of disease progression, wherein cells implanted in the pons spread from a localized lesion to the cerebellum after a period of rapid growth, and without visible enhancement [12, 13]. Analysis of the resected brain by histopathology confirmed viable DIPG in the pons, along with the associated cerebellar infiltration, which supported findings from metabolic imaging indicating temporal changes.

Because DIPG is radiographically characterized by poorly perfused and faintly enhancing heterogeneous lesions, its diagnostic assessment remains challenging. In this context, the relative elevation of lactate in nonenhancing lesions compared to healthy control tissue was a defining feature that may hold diagnostic value for patients as an *in vivo* marker of disease. As these tumors frequently display high levels of LDHA [14, 15] that preferentially convert pyruvate to lactate, imaging of hyperpolarized [1-^13^C]-pyruvate might offer a targeted means of monitoring tumor growth and disease status. The nuclear polarization techniques implemented here provided sufficient signal enhancement to detect real-time pyruvate-to-lactate conversion in the brainstem with high sensitivity, as well as distinguish longitudinal variation in metabolism from growing tumor over relatively brief intervals. Based on the quality and spatial resolution of the ^13^C spectra achieved via hardware and sequence performance, it was possible to evaluate metabolic differences between T_2_-hyperintense lesions and contralateral brain tissue.

Although our focus was on demonstrating feasibility, we believe that a promising application of this technique may be monitoring response to treatment in patients with DIPG, given the inadequacy of conventional MR imaging. By administering [1-^13^C]-pyruvate as a hyperpolarized substrate with measurable conversion to [1-^13^C]-lactate, studies have already managed to provide evidence for both localization of malignant tissue and treatment-induced reduction of metabolic activity arising from growth arrest or apoptosis [16, 17]. A recent study has demonstrated the first application of hyperpolarized ^13^C MR metabolic imaging in patients with supratentorial glioma and presented the safety and feasibility of using hyperpolarized [1-^13^C]-pyruvate to evaluate *in vivo* brain metabolism [18]. Perhaps the greatest technical challenge to translating hyperpolarized imaging to the clinic for diffusing intrinsic pontine glioma is ensuring adequate SNR in the brainstem, where the surrounding tissue is less perfused and far removed from coil elements.

While several single- and multivoxel ^1^H spectroscopy studies have indicated that ^1^H magnetic resonance spectroscopy (MRS) may be useful for assessing disease progression and monitoring response to treatment [19–22], the acquisition of proton spectra in the infratentorial region is generally challenging due to susceptibility effects around the brainstem region and confounded by overlapping lipid peaks that reflect contamination from the surrounding skull. The susceptibility effects encountered in ^13^C MR are considerably smaller relative to ^1^H MR by virtue of the ^13^C gyromagnetic ratio, which is one-fourth that of ^1^H. The proposed method of assessing real-time metabolism using hyperpolarized ^13^C MRSI, combined with anatomical MRI and ^1^H MRS, may provide complementary information that is of value in assessing disease status and response to treatment in DIPG.

Interestingly, the ratio of lactate-to-pyruvate in the nonenhancing T_2_ lesion from this study (0.70 ± 0.24) was significantly smaller than that of enhancing tumor from supratentorial orthotopic glioblastoma xenografts in a previous study (1.0 ±0.36) (*p* < 0.02, unpaired *t*-test), while it remained similar between contralateral brainstem tissue (0.28 ± 0.11) and contralateral supratentorial brain tissue (0.29 ± 0.17) [11]. Future studies will attempt to elucidate the molecular and pathologic mechanisms that produce different characteristics in pyruvate metabolism depending on the type of glioma.

## 4. Conclusions

The results from this study characterized pyruvate and lactate metabolism in orthotopic DIPG xenografts and suggest that hyperpolarized ^13^C pyruvate MRSI is a promising noninvasive imaging tool for the *in vivo* monitoring of biochemical processes in DIPG.

## Figures and Tables

**Table 1:** Summary of ^13^C metabolite quantification. Metabolite values are mean ± SD.

	Lactate/pyruvate^a,b^	Lactate/total carbon^a,b^	Normalized lactate^a,b^	Normalized pyruvate
T_2_-hyperintense lesion (*n*=8)	0.70 ± 0.24	0.36 ± 0.08	2.9 ± 1.1	1.0 ± 0.29
Contralateral brain (*n*=8)	0.28 ± 0.11	0.18 ± 0.07	1.1 ± 0.33	1.1 ± 0.35
Healthy rat brain (*n*=5)	0.20 ± 0.06	0.14 ± 0.03	1.2 ± 0.25	1.1 ± 0.25

^a^Significant difference between T_2_-hyperintense lesion and contralateral brain (*p* < 0.001). ^b^Significant difference between T_2_-hyperintense lesion and healthy rat brain (*p* < 0.002).

## Data Availability

The data used to support the findings of this study are available from the corresponding author upon request.
